# Comparison of outcomes between indirect decompression of oblique lumbar interbody fusion and MIS-TLIF in one single-level lumbar spondylosis

**DOI:** 10.1038/s41598-021-92330-9

**Published:** 2021-06-17

**Authors:** Shih-Feng Hung, Jen-Chung Liao, Tsung-Ting Tsai, Yun-Da Li, Ping-Yeh Chiu, Ming-Kai Hsieh, Fu-Cheng Kao

**Affiliations:** 1 Department of Biomedical Engineering and Environmental Sciences, National Tsing Hua University, Hsinchu, Taiwan; 2 Department of Orthopaedic Surgery, Spine Section, Bone and Joint Research Center, Chang Gung Memorial Hospital and Chang Gung University College of Medicine, No. 5, Fusing St., Gueishan, Taoyuan Taiwan

**Keywords:** Neuroscience, Health occupations, Neurology

## Abstract

Minimal invasive spinal fusion has become popular in the last decade. Oblique lumbar interbody fusion (OLIF) is a relatively new surgical technique and could avoid back muscle stripping and posterior complex destruction as in minimally invasive transforaminal lumbar interbody fusion (MIS-TLIF). Between December 2016 and September 2018, patients with single level degenerative spondylosis were selected to enroll in this retrospective study. A total of 21 patients that underwent OLIF and 41 patients that received MIS-TLIF were enrolled. OLIF showed significantly less blood loss and shorter surgery time compared to MIS-TLIF (p < 0.05). The improvement in segmental lordosis and coronal balance was significantly more in OLIF group than MIS-TLIF group (p < 0.05). When comparing with MIS-TLIF, OLIF was significantly better in Oswestry Disability Index (ODI) and visual analogue scale for back pain improvement at post-operative 6 months (p < 0.05). Both OLIF and MIS-TLIF are becoming mainstream procedures for lumbar degenerative-related disease, especially for spondylolisthesis. However, the indirect decompression of OLIF has shown to have less perioperative blood loss and shorter surgery time than that of MIS-TLIF. In addition, OLIF gives superior outcome in restoring segmental lordosis and coronal imbalance. While both OLIF and MIS-TLIF provide optimal clinical outcomes, upon comparison between the two techniques, the indirect decompression of OLIF seems to be a superior option in modern days.

## Introduction

Many adults have experienced lower back pain at some point during their lifetime, and most of these symptoms resolve or improve without intervention within a few weeks. However, for a small number of these patients, lumbar fusion surgery may be required, especially when patients experience concomitant leg pain or deformity of lumbar body is present. Lumbar interbody fusion surgery is a treatment for lumbar spine-related disease including degenerative disc disease, spondylolisthesis, and disc herniation. The main objective of this technique is to create interbody fusion between two vertebral bodies in order to achieve a stable, decompressed vertebral structure.

A variety of techniques of lumbar interbody fusion have been used to help alleviate pain from lumbar spine-related disease^1–3^. For the past few decades, the minimally invasive spinal surgical technique has emerged as an alternative approach to the more invasive open lumbar interbody fusion procedure^4–6^. The minimally invasive technique for transforaminal lumbar interbody fusion (MIS-TLIF) was first introduced in 2002, and it has since helped improve several perioperative outcomes including operative bleeding, infection incidence, surgical injury, and days of discharge^7–10^.

Another technique called oblique lateral interbody fusion (OLIF) has been becoming more popular in the twenty-first century. It offers solution to the potential surgical traumas caused by other conventional techniques by accessing lumbar disc through space between the aorta and psoas muscle^11^. This retroperitoneal lumbar approach, which was first coined in 2012, helps achieve indirect decompression and preserve integrity of posterior column structure^12^.

Since OLIF is still a relatively new technique, there are only few studies that compare MIS-TLIF and OLIF based on their clinical and radiologic outcomes. The purpose of this study is to compare the radiographic and clinical outcomes between single-level OLIF with lateral cortical screw (LCS) fixation and MIS-TLIF in the patients with degenerative lumbar spondylosis.

## Method

### Study population and surgical techniques

Between December 2016 and September 2018, a retrospective review was conducted from the spine department in our institution to identify all patients that underwent either OLIF or MIS-TLIF. All the OLIF cases were performed by a single surgeon from December 2016 to September 2018, and all the MIS-TLIF cases were done by another individual surgeon during the same period of time. Inclusion criteria for this study included patients with single lumbar level lesion (L2–L5) and had either degenerative spondylolisthesis, disc degeneration with vacuum phenomenon, neurogenic intermittent claudication, clinical sciatica, or mechanical low back pain with segmental instability. Exclusion criteria included spinal infection, spinal fracture, benign or malignant spinal tumor, revision surgery at the same level, and cervical or thoracic lesions, and any patients with less than 2 years of follow-up.

**Figure 1:**
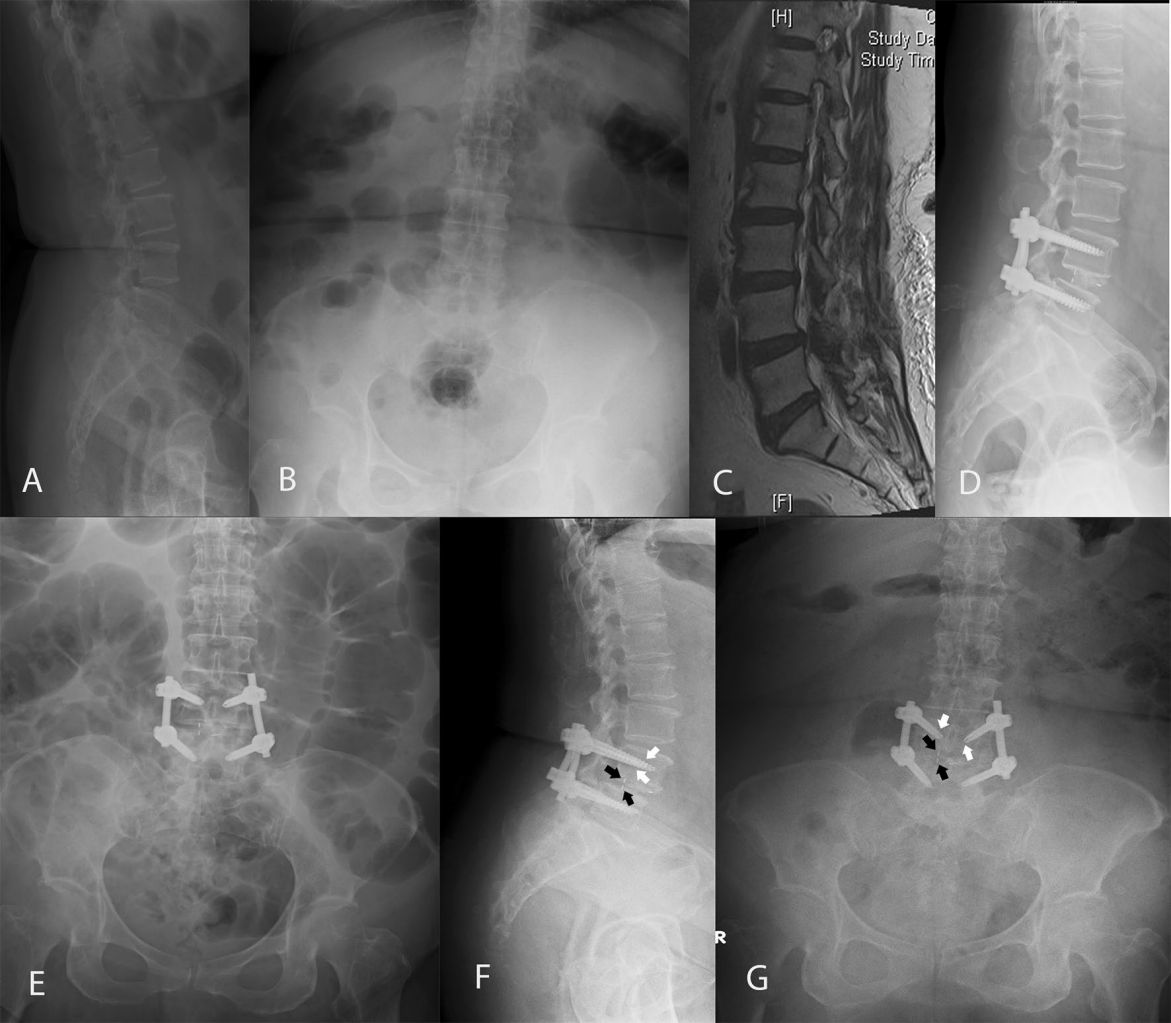
A patient was diagnosed preoperatively with degenerative spondylolisthesis and lumbar spinal stenosis at L4-5 level, and underwent minimally invasive transforaminal lumbar interbody fusion (MIS-TLIF). Radiographic images (**A**,**B**) and MRI (**C**) were taken preoperatively. The immediate post-operative lumbar spine lateral view (**D**) and lumbar antero-posterior view (**E**) are shown. Cage subsidence (black arrow) and screw halo sign (white arrow) were seen on the last follow-up images (**F**,**G**).

**Figure 2:**
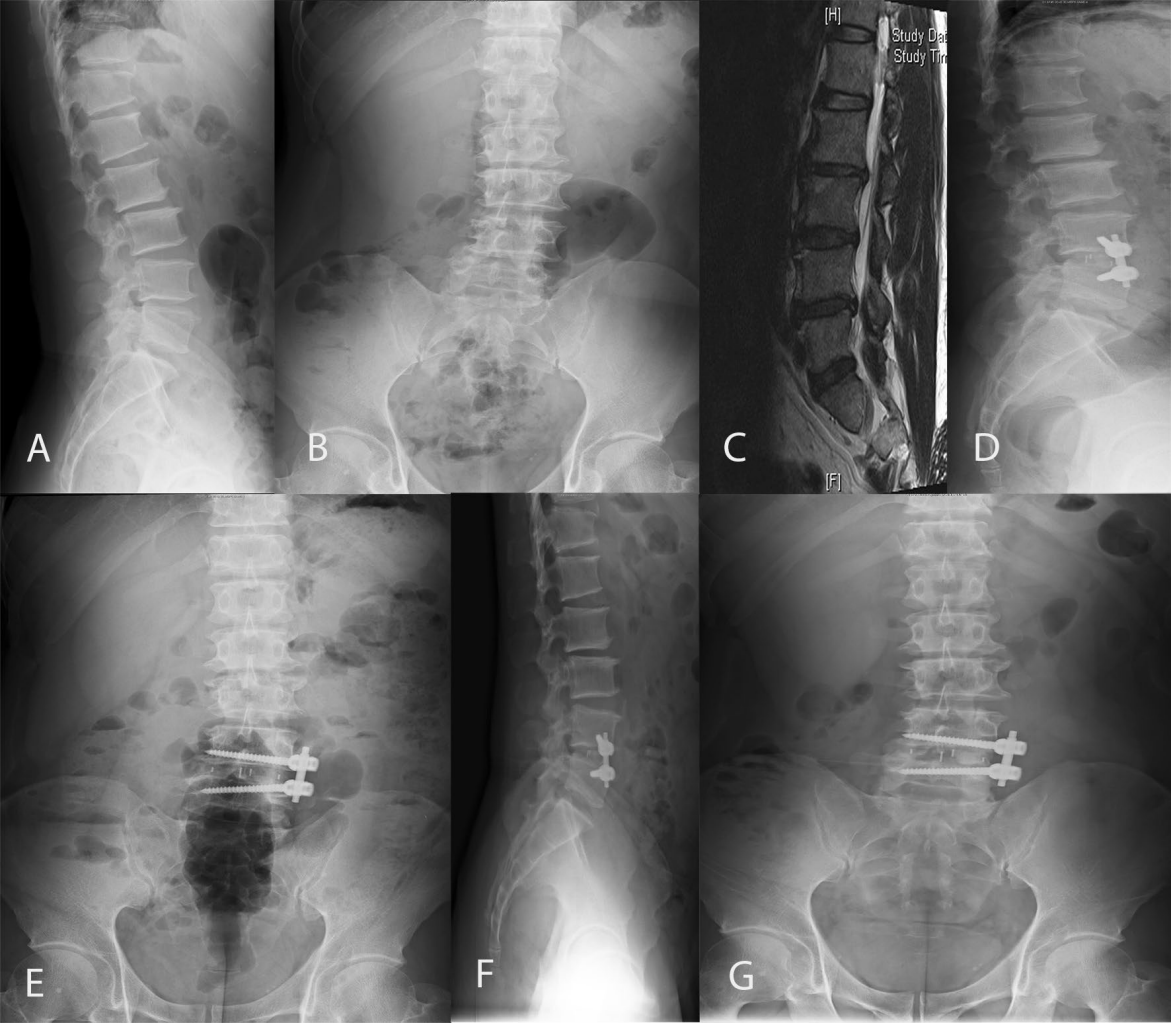
A patient was diagnosed with degenerative spondylolisthesis and lumbar spinal stenosis at L4-5 level, as shown on lumbar spine lateral view (**A**), lumbar antero-posterior view (**B**), and T2-weighted MR image (**C**). He received oblique lumbar interbody fusion (OLIF) at L4-L5 with lumbar spine lateral view (**D**) and lumbar antero-posterior view images (**E**) taken immediately after the surgery. Follow-up images (**F**,**G**) were taken at the last follow-up.

For the MIS-TLIF group, the patient was placed on a 4-postspinal frame in the prone position under general anesthesia. Surgical level was identified using portable (C-arm) radiography prior to the procedure. Depending on the patients’ symptom or severity of spinal stenosis from preoperative image studies, unilateral Wiltse paraspinal approach was performed on the side to achieve further transforaminal lumbar interbody fusion^13^. Skin, soft tissue and back muscles were retracted by tubular expandable retractors to expose the facet joint. Hemilaminectomy and facetectomy were then made to well decompress the nerve roots. The bone removed was kept and used as autograft for transforaminal lumbar interbody fusion. After nerve decompression and endplate preparation were done, a bullet-shaped cage, lordotic angle 4º, filled with autograft bone and demineralized bone matrix was inserted with standard TLIF techniques. Cage height was equal to or larger than preoperative disc height on plain radiography to make sure there was no local motion during operation. The entire procedure was carried out under a surgical microscope with variable magnification and focalization. After completing MIS-TLIF, bilateral percutaneous pedicle screws were then applied (Fig. 1). On the other hand, the patients in the OLIF group were placed in lateral decubitus position with left side up. Breaking operation table to bend lumbar spine was not required. It would be advisable for the surgeon to obtain true lateral and anteroposterior views by checking portable (C-arm) radiography before the procedure started. Then, a surgical incision of 4–6 cm and 3–5 cm ventral to the anterior vertebral cortex of the target intervertebral disc was made. We used blunt dissection of abdominal external oblique, internal oblique, and transversalis muscles to approach the retroperitoneal space and the disc space in the oblique corridor (the space between the major blood vessel and the psoas muscle)^14^. After the target disc space was identified, sequential soft tissue dilators and blade retractor were applied to establish the retroperitoneal pathway to the oblique corridor under direct visualization. Mild retraction and splitting anterior part of the psoas muscle would minimize the potential risk of injuring the motor nerves and be performed depending on the anatomic structures of each patient^15^. The cage filled with cancellous allograft bone was then inserted after well anterior discectomy and endplate preparation. The cage size that was larger than pre-operative disc height was chosen to assure the augmentation in neuroforamen after the cage insertion. Indirect decompression was achieved through disc height restoration and neuroforamen expansion. Lateral instrumentation by LCS fixation was applied at the oblique corridor close to the endplates (Fig. 2).

All patients included in this study had completed 2 follow-ups during the first 6 months (interval of 3 months) and then every half a year after for a minimum of 2 years. Other parameters recorded included age, sex, BMI, surgery time, blood loss, days of discharge, history of smoking, and history of diabetic mellitus. Medical records, laboratory data, radiographic images, and functional data of these patients were reviewed and analyzed. This study was approved by Chang Gung Medical Foundation Institutional Review Board (IRB), and the requirement for informed consent was waived due to the retrospective nature of the study. All procedures performed in this study were in accordance with the ethical standards of the national research committee.

### Clinical functioning

Functional data for clinical outcomes were evaluated using visual analog scale (VAS) to assess lower back pain, leg pain, soreness, and the Oswestry Disability Index (ODI). The VAS evaluations were done at pre-operation, post-operation (3 days after surgery), and 6-months follow-up. The ODI assessments were done at pre-operation and 6-months follow-up.

### Radiography

**Figure 3:**
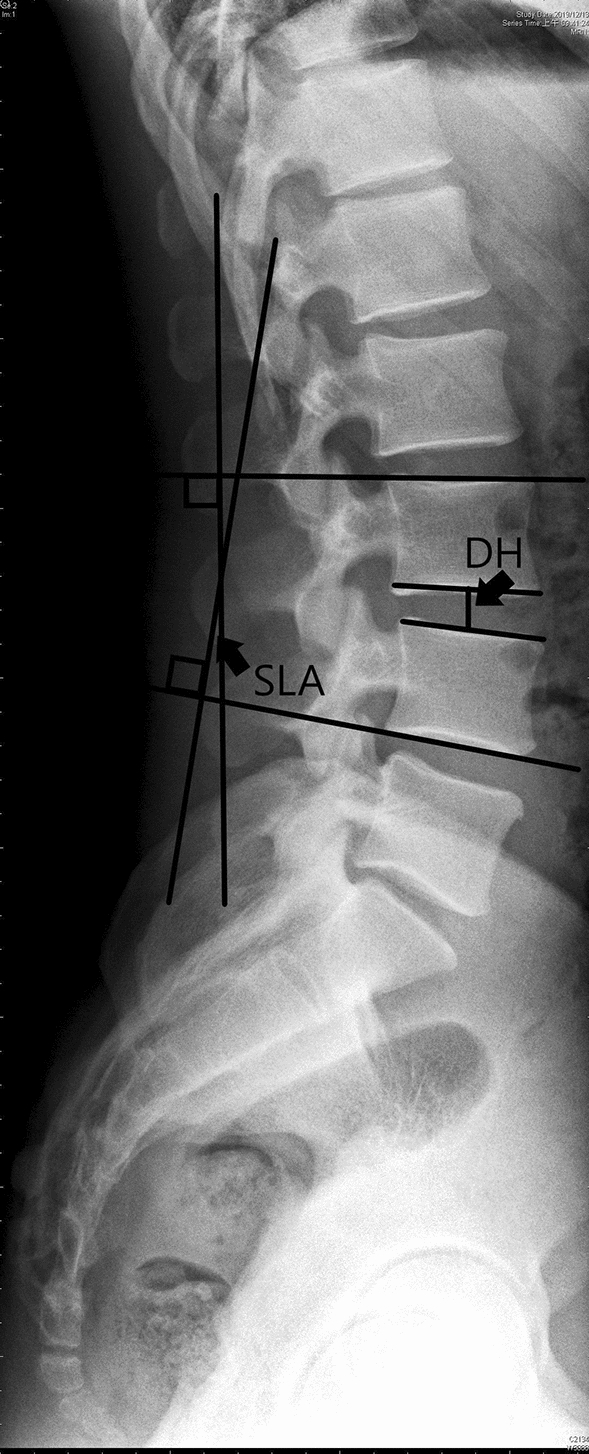
The lumbar spine lateral view image of a patient without any underlying disease was used to demonstrate how the segmental lordotic angle and disc height were measured. Segmental lordotic angle (SLA) is defined as the angle subtended by the superior endplate line of upper vertebral body and the lower endplate of lower vertebral body. Disc Height (DH) is defined as the distance from mid-position of upper endplate to lower endplate of two vertebral body.

**Figure 4:**
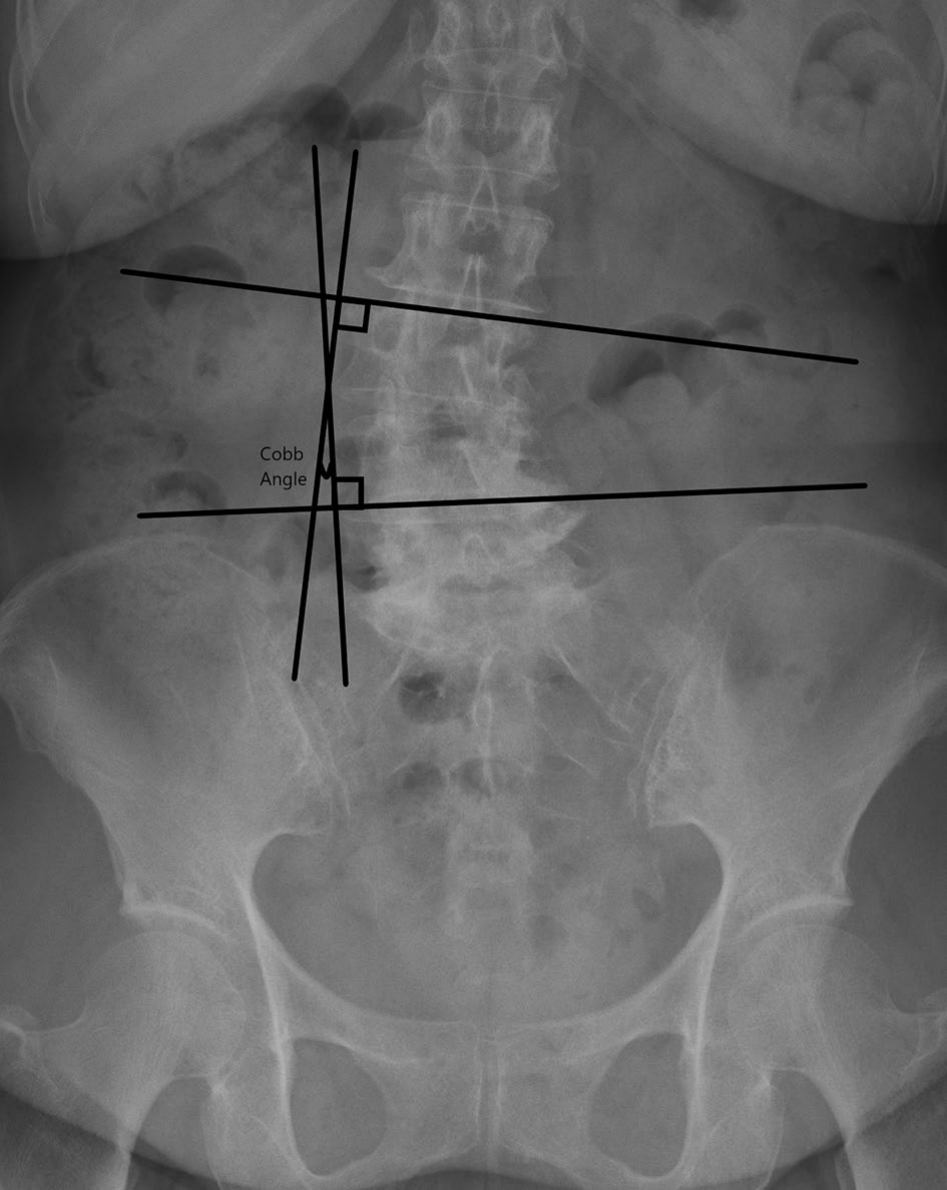
The measurement of coronal tilting angle was assessed by measuring the Cobb angle of superior endplate line of upper vertebral body and the lower endplate of lower vertebral body on lumbar antero-posterior view image.

Radiographs of the patients were taken during pre-operation, immediate post-operation (3 days after surgery), and follow-ups. Measurements were taken for segmental lordotic angle (SLA), disc height (DH), cage subsidence (CS), screws halo sign (SHS), and coronal tilting angle (CTA). A single investigator measured SLA, DH, CTA, and their changes between pre-operation, post-operation, and follow-ups. SLA was obtained via lateral view of lumbar spine x-ray image by measuring the angle between upper end plate of vertebral body to lower endplate of lower vertebral body (Fig. 3). DH was defined as the distance from mid-position of upper endplate to lower endplate of two vertebral body (Fig. 3). CTA was assessed by measuring the Cobb angle of plane radiography of lumbar antero-posterior view (Fig. 4). The evaluations for SHS and CS were assessed at the end of the 24 months follow-up by plane radiography of lumbar as well (Fig. 1F,G). CS was evaluated by measuring whether the cage migration was greater than 2-mm. Solid fusion status was defined as trabecular bone formation without gap between the vertebral endplate and the cage in the sagittal or coronal planes on plain x-rays; it was evaluated by at the 12 and 24- months follow-up.

### Statistical analysis

Statistical calculations were performed using SPSS 25.0 to analyze the parameters in both groups of patients. All quantitative variables were presented as mean standard deviation, and qualitative variables were shown in terms of ratio and number. Continuous variables were evaluated by Student’s t-test. The categorical variables were performed by Fisher exact test and Mann–Whitney test. A P value < 0.05 was considered to be statistically significant difference.

## Result

From December 2016 to September 2018, a total of 27 patients that underwent one single-level OLIF and 57 patients that received one single-level MIS-TLIF were selected. In the MIS-TLIF group, 11 patients were excluded from this study due to the surgical level including sacrum, and another 5 were due to the loss of follow-up. From the OLIF group, 3 patients were excluded because they had less than 2 years of follow-up and another 3 were excluded because they previously received either laminotomy or discectomy at the same level. After the screening, a total of 41 MIS-TLIF patients and 21 OLIF patients were selected to enroll in this study.

The demographics for OLIF and MIS-TLIF groups were comparably matched. The mean ages (years) for MIS-TLIF and OLIF were 60.32 and 62.33, respectively (p = 0.5634). The mean BMI’s (kg/m2) for MIS-TLIF and OLIF were 26.25 and 26.37, respectively (p = 0.9179). Among the 41 MIS-TLIF patients, 8 of them had smoking history compared to 4 (out of 21) from the OLIF group (p = 0.965). Eight patients from the MIS-TLIF group had history of diabetic mellitus while the OLIF group had 4 patients (p = 0.965). The MIS-TLIF group had 27 patients were diagnosed with degenerative spondylolisthesis, 5 with herniated intervertebral disc, and 29 with lumbar spinal stenosis; the OLIF group had 14 patients with degenerative spondylolisthesis, 2 with herniated intervertebral disc, and 15 with lumbar spinal stenosis (p = 0.8657, 0.8095, 0.9159, respectively). In the MIS-TLIF group, 20 of the patients had surgery level at L4–5, 19 patients at L3–4, and 2 patients at L2–3; in the OLIF group, 12 patients received the treatment at L4–5, 8 of them at L3–4, and 1 patient at L2–3 (p = 0.8001, 0.8812, 0.9257, respectively) (Table 1).

**Table 1:** Demographic parameters and the comparisons of the two surgical techniques.

	MIS-TLIF	OLIF	p-value
Number of patients	41	21	
Age	60.32 ± 13.34	62.33 ± 12.08	0.5634
Sex (Male: Female)	28:13	10:11	0.1137
Smoker	8	4	0.965
BMI (kg/m^2^)	26.25 ± 3.96	26.37 ± 4.43	0.9179
Diabetic Mellitus	8	4	0.965
**Surgical techniques**			
Patient position	Prone on a 4-postspinal frame	Lateral decubitus position with left side up	
Surgical approach	Unilateral Wiltse paraspinal	Oblique retroperitoneal	
Decompression method	Direct decompression via hemilaminectomy and facetectomy	Indirect decompression via disc height restoration and neuroforamen expansion	
Fixation	Posterior percutaneous pedicle screws	Lateral instrumentation by pre-psoas bil-cortex pedicle screw fixation	

Values are mean ± standard deviation; OLIF: oblique lateral lumbar interbody fusion; MI-TLIF: minimally invasive transforaminal lumbar interbody fusion; BMI: body mass index.

*p < 0.05, statistical significance.

The average surgery time for OLIF was significantly shorter than that of MIS-TLIF (93.95 ± 14.84 vs 136.38 ± 31.18, p = 0.0291). The patients that received OLIF had fewer days of hospital discharge compared to the MIS-TLIF patients (4.05 ± 1.56 vs 6.39 ± 1.41, p < 0.001). Blood loss during surgery was also significantly less in OLIF than in MIS-TLIF (90.48 ± 19.74 ml vs 167.32 ± 35.93 ml, p = 0.0406) (Table 3).

### Functional outcomes

Using the VAS pain score, the mean pre-operative back pain scores were relatively comparable between OLIF and MIS-TLIF (7.00 ± 1.26 vs6.61 ± 1.66, p = 0.3563) (Table 2). The post-operative back pain scores were 1.05 ± 0.94 for OLIF and 7.56 ± 1.18 for MIS-TLIF (p < 0.0001). There was significant difference in back pain score between OLIF and MIS-TLIF for the 6-months follow up (0.75 ± 1.02 vs 3.95 ± 1.53, p < 0.0001). Pre-operative leg pain scores for OLIF and MIS-TLIF were 6.00 ± 1.21 and 6.32 ± 2.01, respectively (p = 0.4475); post-operative leg pain scores were 0.75 ± 0.72 and 0.85 ± 0.88 (p = 0.6496). The preoperative ODI’s were similar between the 2 groups (OLIF: 66.35 ± 5.97, MIS-TLIF: 69.71 ± 7.24, p = 0.6532). The ODI improvement in follow-ups between the 2 groups showed significant difference (OLIF: 33.30 ± 12.77, MIS-TLIF: 21.51 ± 9.94, p = 0.0260).

**Table 2:** Perioperative and clinical outcomes.

	MIS-TLIF	OLIF	p-value
Blood loss	167.32 ± 35.93	90.48 ± 19.74	0.0406*
Surgery time	136.38 ± 31.18	93.95 ± 14.84	0.0291*
Days of discharge	6.39 ± 1.41	4.05 ± 1.56	< 0.0001*
Pre VAS back pain	6.61 ± 1.66	7 ± 1.26	0.3563
Post VAS back pain	7.56 ± 1.18	1.05 ± 0.94	< 0.0001*
F/U VAS back pain	3.95 ± 1.53	0.75 ± 1.02	< 0.0001*
Pre VAS leg pain	6.32 ± 2.01	6 ± 1.21	0.4475
Post VAS leg pain	0.85 ± 0.58	0.75 ± 0.62	0.6496
Pre ODI	69.71 ± 7.24	66.35 ± 5.97	0.6532
Follow-up ODI	48.2 ± 5.93	33.05 ± 9.96	< 0.0001*
ODI improvement	21.51 ± 9.94	33.30 ± 12.77	0.0260*

Values are mean ± standard deviation; ODI: Oswestry Disability Index; VAS: visual analogue scale; Post: pos-operative (3 days after surgery); F/U: follow-up (6 months after surgery).

*p < 0.05, statistical significance.

### Radiographic outcomes

The SLA increased from a mean of 14.75 preoperatively to 18.40 postoperatively in OLIF and MIS-TLIF showed a decrease from a mean of 17.12 preoperatively to 12.71 postoperatively (Table 3). There was significant difference in the immediate SLA change between OLIF and MIS-TLIF (3.65 ± 4.98 vs -4.41 ± 7.94, P = 0.0098). The follow-up SLA decreased in both OLIF and MIST-TLIF (13.13 ± 6.7 vs 11.43 ± 5.89) and the difference in change was close to statistical significance (P = 0.0504). The CTA for OLIF was 6.62 ± 4.01 preoperatively, 1.93 ± 1.48 postoperatively, and 3.13 ± 2.38 follow-up; MIS-TLIF had CTA of 4.02 ± 2.08 preoperatively, 2.24 ± 0.77 postoperatively, and 2.81 ± 0.92 follow-up. Coronal tilting angle change (CTAC) did indicate a significant difference in both the immediate postoperative CTAC and follow up CTAC (p = 0.0019, 0.00014, respectively) between the 2 groups. The immediate disc height change (millimeters) was 3.44 ± 1.41 in OLIF and 2.99 ± 1.53 in MIS-TLIF. The follow up disc height changes (millimeters) for OLIF and MIS-TLIF were 2.34 ± 1.05 and 1.96 ± 1.02, respectively. Both the immediate disc height change and follow-up disc height change between the 2 groups showed no significant difference (p = 0.2567, 0.3152, respectively). For fusion status, 16 out of 21 OLIF patients and 26 out of 41 MIS-TLIF patients had complete union at 12-months follow-up (p = 0.3120); 19 OLIF patients and 36 MIS-TLIF patients showed complete union at the end of 24-monthsfollow-up (p = 0.7854). Lastly, 5 out of 21 OLIF patients and 11 out of 41 MIS-TLIF patients showed post-operative CS and SHS (p = 0.7956).

**Table 3:** Radiographic outcomes.

	MIS-TLIF	OLIF	p-value
Pre SLA	17.12 ± 8.73	11.75 ± 6.18	0.1014
Post SLA	12.71 ± 5.38	15.4 ± 7.53	0.1093
F/U SLA	11.43 ± 5.89	13.13 ± 6.7	0.3411
Post SLA change	−4.41 ± 7.94	3.65 ± 4.98	0.0098*
F/U SLA change	−5.69 ± 8.19	1.38 ± 4.54	0.0504
Pre CTA	3.02 ± 2.08	6.62 ± 4.01	0.0007*
Post CTA	1.24 ± 0.77	1.93 ± 1.48	0.0583
F/U CTA	1.81 ± 0.92	3.13 ± 2.38	0.0350*
Post CTA change	−1.78 ± 1.13	−4.69 ± 3.59	0.0019*
F/U CTA change	−1.21 ± 1.87	−3.49 ± 3.03	0.0014*
Pre disc height	7.38 ± 1.76	7.08 ± 1.77	0.5255
Post disc height	10.37 ± 1.11	10.52 ± 1.4	0.6316
F/U disc height	9.34 ± 1.12	9.42 ± 0.81	0.7894
Post disc height change	2.99 ± 1.53	3.44 ± 1.41	0.2567
F/U disc height change	1.96 ± 1.02	2.34 ± 1.05	0.3152
Pre NFH (mm)	17.66 ± 2.895	16.76 ± 2.278	0.168
Post NFH (mm)	20.49 ± 3.171	22.24 ± 2.071	0.008*
NFH improvement (%)	17.9 ± 21.2	34.8 ± 21.0	< 0.001*
**Post cage subsidence**			
Yes	11	5	0.7956
No	30	16	
**Post halo sign**			
Yes	11	5	0.7956
No	30	16	
**Fusion status**			
Solid	26	16	0.3120
Incomplete	15	5	

Values are mean ± standard deviation; SLA: segmental lordotic angle; CTA: coronal tilting angle; Post: post-operative (3 days after surgery); F/U: final follow-up (24 months after surgery); NFH: neuroforaminal height.

*p < 0.05, statistical significance.

## Discussion

OLIF and MIS-TLIF take different approaches. MIS-TLIF introduces direct spinal decompression through laminectomy and involves paraspinal access from posterolateral side of the lumbar intervertebral disc, which requires to some extent retraction of nerve roots and spinal erector muscles; however, major vessels like aorta can be unharmed. The posterior pedicle screw fixation is then used to provide mechanical support for the spine. On the other hand, OLIF accesses the space of lumbar disc by entering through the anatomical space between psoas muscle and aorta artery; one of the disadvantages of OLIF is iatrogenic vascular injury. OLIF mainly uses indirect decompression to alleviate compressed neural element, and many recent studies have shown promising results in anterolateral lumbar interbody fusion approach.

We first compared the intraoperative data between OLIF and MIS-TLIF groups. Several literatures have pointed out that prolonged surgery time is associated with increased operative complication, and shorter operation time has positive impact on postoperative outcome. Less perioperative blood loss is also beneficial to patients, including reduced risk of exposure to pathogens, blood transfusion complication, perioperative anemia, morbidity, and mortality. In a recent systemic review on OLIF by Li et al., the 16 selected literatures showed a mean blood loss of 109.9 ml and an average surgery time of 95.2 min^16^. Another retrospective study on MIS-TLIF with 20 patients enrolled by Lee et al. reported an average surgery time of 131.7 min and 208.3 ml blood loss^17^. In our study, the average surgery time and perioperative blood loss for OLIF was significantly less than that of MIS-TLIF. While the surgery time and blood loss can vary depending on surgeon’s expertise and repertoire, several studies have suggested that OLIF has superior perioperative results when comparing to MIS-TLIF. The OLIF’s anterolateral approach allows surgeons to access surgical site through a small incision on the left lower abdomen where there are only few muscle layers that need to be retracted. Moreover, bony structural damage is avoided since laminectomy is not required in OLIF. The decrease in incidence of iatrogenic disturbance to the surrounding tissues and nerves ultimately yields better outcome in surgical bleeding and surgery time^18,19^. Consequently, OLIF provides patients with better post-operative recovery and reduced risk of surgical complication.

Back muscles play vital roles in connecting multiple major muscles of human body parts, and OLIF allows back muscles to remain intact after surgery. By leaving the paraspinal muscles unharmed and with less soft tissue traction, patients are likely to have better post-operative recovery and shorter hospital stay. A retrospective study by Ohtori et al. investigated 35 patients that received OLIF, and the results demonstrated its effectiveness, with an average of 34 points improvement in ODI, 6.7 and 3.2 points decrease in VAS of leg and back, respectively^20^. According to several studies, a change of at least 15 points in ODI score and at least 3.5 points improvement in VAS can be an indicator to excellent operative outcome^21,22^. In our study, the average VAS pain scores and ODI reported by the patients showed clinically significant improvement in both groups. However, when comparing the outcome of the 2 groups, OLIF had the upper hand in the post-operative back pain improvement and days of hospital discharge. It is worth noting that 8 of the 21 OLIF patients experienced lower limb weakness and front thigh numbness on the same side where the procedure took place during the first month. However, only 2 of those OLIF patients continued to feel numbness at 6 months follow-up. This phenomenon was likely due to genitofemoral nerve disturbance during the procedure, in which the disturbance is usually temporary and reversible. On the other hand, 3 MIS-TLIF patients reported lower limb pain after the surgery and at 6 months follow-up. No other complication such as arterial injury or dural tear was reported from the 2 groups.

The sagittal balance is linked to better alignment of vertebral spine. The restoration of lumbar lordosis and disc height has been associated with better postoperative clinical outcomes. According to Videbaek et al., 92 patients with severe lower back pain were chosen for either posterolateral lumbar interbody fusion or anterior lumbar interbody fusion^23^. The sagittal balance parameters were analyzed. The result showed patients who had restored their sagittal balance had a significantly better clinical outcome measured by ODI. In our study, the OLIF group showed improvement in restoring segmental lordosis while the MIS-TLIF group had a decrease in segmental lordosis post-operatively. The reason for the decrease in segmental lordosis in the MIS-TLIF group was not clear, as patient-surgery interplay of multiple factors might have been involved, such as the bullet-shaped cage that was used in MIS-TLIF. For instance, in a study published by Gödde et al. a total of 42 patients that underwent posterior lumbar interbody fusion^24^. Twenty patients inserted with bullet-shaped cage had a mean decrease in segmental lordosis from 10° to 2° at L3–L4 and from 10°to 5°at L4–L5 while 22 patients inserted with wedge-shaped cage had an average increase in segment lordosis from 4° to 7° at L3–L4 and 2° to 8° at L4-L5. The cage geometry likely plays a role in alignment of lumbar spine after lumbar interbody fusion. There are several factors that can lead to kyphotic deformity at the fused segment after TLIF is done, including distraction due to pedicle screw insertion and the relative posterior location of the cage^25^. In addition, many studies on surgical outcomes of lumbar interbody fusion have suggested that having a cage covered in the anterior part of vertebral body could help with restoration of lumbar lordosis since the anterior portion of endplate is the strongest part^26–28^. This theory aligns with our result, as OLIF, with its wide cage being inserted in the relatively anterior part of vertebral body, showed better improvement in restoring segmental lordosis post-operatively.

The cage’s relative anterior location in OLIF also helps provide faster fusion rate by correcting sagittal alignment, less endplate damage, and better mechanical support^29^. Our study indicated that the OLIF group had better fusion rate at 12-months follow-up, although not statistically significant; the fusion rate at 24-months follow-up was comparable between the 2 groups. Moreover, unlike that of MIS-TLIF, the pedicle screws in OLIF are used only for cage fixation rather than direct mechanical support. Even though our results showed no difference between the 2 groups in cage subsidence and screw halo sign incidence, theoretically speaking, the fact that OLIF uses wider cage and pedicle screws that don’t bear as much stress as that of MIS-TLIF means OLIF patients could have less chance of cage-subsidence and screw halo sign post-operatively.

Coronal imbalance is not seen as a major surgical indication in most literatures. However, patients suffering from coronal imbalance tend to have increased pain, discomfort, and decreased the quality of daily life^30^. It is because, unlike sagittal malalignment, there are not many compensatory mechanisms available for coronal malalignment in a human body. Some studies have shown that coronal tilting angle can be corrected by oblique lumbar interbody fusion technique. In a study by Patel et al., 15 patients underwent OLIF and radiologic evaluation was done^31^. Cobb’s angle, lumbar lordosis, sagittal vertical axis, thoracic kyphosis, sacral slope, pelvic tilt, and pelvic incidence all showed significant correction immediately after the procedure. All the parameters except thoracic kyphosis were maintained at the last follow-up. In another study by Wang et al., 11 patients with lumbar degenerative disease underwent a combination of OLIF and lateral fixation^32^. Radiographic results showed significant improvement in coronal Cobb angle from 15.3° pre-operatively to 5.9° post-operatively. OLIF procedure is capable of restoring the global and sagittal spinal alignment via leveling the vertebrae and disc space intended to fuse. Furthermore, Temple et al. suggested that lateral fixation with pre-bent rod could accomplish further sagittal and coronal plane correction^33^. In our study, while both groups showed improvement in coronal tilting angle, the OLIF group showed significant difference in reduction of coronal tilting angle in post-operation and follow-ups compared to that of MIS-TLIF. Like previously mentioned, MIS-TLIF uses a smaller bullet-shaped cage that is inserted in the central part of the vertebral body. On the other hand, OLIF uses a larger cage that is inserted in the anterior part of the vertebral body, which gives stronger and more evenly support to the spine. This may explain the edge that OLIF had over MIS-TLIF in coronal balance correction in our study.

To the best of our knowledge, our study is the first to investigate the efficiency of indirect decompression in OLIF with LCS fixation, comparing the clinical and radiographic outcomes with MIS-TLIF. Some studies have proven indirect decompression of OLIF could attain sufficient nerve release and preserve the posterior column structures^19,29,34–36^. The others illustrated how OLIF with LCS is a safe and effective procedure with adequate clinical outcome and deformity correction^32,37,38^. Patients undergoing indirect decompression of OLIF with LCS in our study felt equivalent postoperative leg pain relief as them in the MIS-TLIF group. Our OLIF group had better postoperative back pain improvement, functional outcome (ODI score) and shorter hospital stay because OLIF with indirect decompression and LCS revealed less blood loss, minimizing surgical time, no posterior column and back muscle violation. All in all, spine surgeons could consider OLIF with LCS as one alternative procedure for minimally invasive lumbar fusion in the future.

There are still some limitations in this study. First of all, it was a retrospective study with relatively small sample size and short-term follow up, so some additional information about the patient groups in this study such as value of bone mass density or lumbar range of motion was not available. In addition, although each surgical technique was done by an individual surgeon, the samples were not randomized, which may have resulted in selection bias. Secondly, each surgical technique was performed by one surgeon. The skillsets and experience of each surgeon could have some impact on the results. Moreover, local damage and retraction time in the OLIF group were not recorded, which could’ve provided some additional insight regarding the patients’ post-operative thigh symptoms. Lastly, the radiographic measurements were done by a single observer, which may have some intra-observer bias.

## Conclusion

Both OLIF and MIS-TLIF are mainstream procedures for lumbar degenerative-related disease. While both OLIF and MIS-TLIF provide optimal clinical outcomes, the indirect decompression of OLIF has shown superior clinical and radiographic outcomes compared to MIS-TLIF. Upon comparison between the two techniques, the anterolateral approach of OLIF seems to be a better option in modern days.

## Data Availability

The datasets generated and/or analysed during the current study are available from the corresponding author on reasonable request.

## Abbreviations

OLIF: Oblique lumbar interbody fusion
MIS-TLIF: Minimally invasive transforaminal lumbar interbody fusion
LCS: Lateral cortical screw
PEEK: Polyetheretherketone
BMI: Body mass index
IRB: Institutional Review Board
VAS: Visual analog scale
ODI: Oswestry disability index
SLA: Segmental lordotic angle
DH: Disc height
CS: Cage subsidence
SHS: Screws halo sign
CTA: Coronal tilting angle
SPSS: Statistical product and service solutions
CTAC: Coronal tilting angle change
